# Supplementary material for: Si metasurface supporting multiple quasi-BICs for degenerate four-wave mixing
Source: Nanophotonics. 2024 Jun 5;13(18):3421–8. doi: 10.1515/nanoph-2024-0128 (PMC11501966; doi:10.1515/nanoph-2024-0128)
Supplement: Supplementary file 1 — Supplementary Material Details [file j_nanoph-2024-0128_suppl_001.pdf]

Supplementary Materials for:

Si Metasurface Supporting Multiple Quasi-BICs for Degenerate Four-Wave Mixing

Gianni Q. Moretti[1,2], *Thomas Weber*[3], *Thomas Possmayer*[3], *Emiliano Cortés*[3], *Leonardo de S. Menezes*[3,4], *Andrea V. Bragas*[1,2], *Stefan A. Maier*[5,6], *Andreas Tittl*[3*], *Gustavo Grinblat*[1,2*]

[1]Universidad de Buenos Aires, Facultad de Ciencias Exactas y Naturales, Departamento de Física, 1428 Buenos Aires, Argentina.

[2]CONICET - Universidad de Buenos Aires, Instituto de Física de Buenos Aires (IFIBA). 1428 Buenos Aires, Argentina.

[3]Chair in Hybrid Nanosystems, Nanoinstitute Munich, Faculty of Physics, Ludwig-Maximilians-Universität München, 80539 München, Germany.

[4]Departamento de Física, Universidade Federal de Pernambuco, 50670-901 Recife-PE, Brazil.

[5]School of Physics and Astronomy, Monash University, Clayton Victoria 3800, Australia.

[6]Department of Physics, Imperial College London, London SW7 2AZ, United Kingdom.

*Email: andreas.tittl@physik.uni-muenchen.de; grinblat@df.uba.ar

Contents

Figure S1: SEM Images of representative fabricated metasurfaces.

Figure S2: Field distributions and multipolar decomposition of all resonances.

Figure S3: Additional transmittance spectra for varying α and d.

Figure S4: Results for different lateral dimensions and simulations changing the height.

Figure S5: Linear and nonlinear experiments with y-polarized light.

Figure S6: Experimental setup for nonlinear measurements.

Figure S7: Ellipsometry data for Si and SiO_2 and simulations changing k.

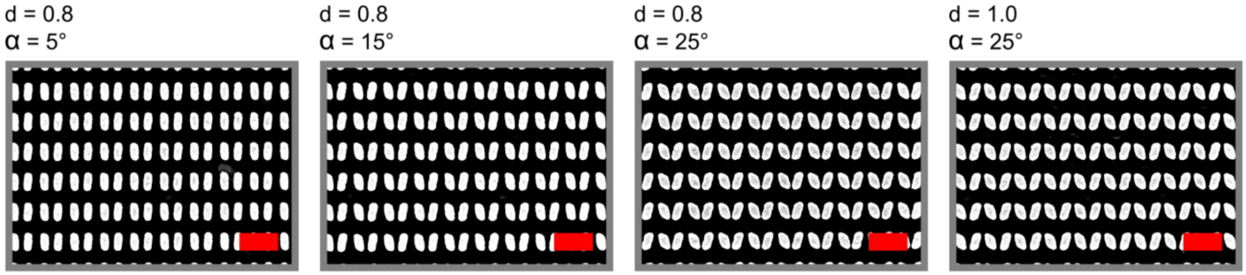

Figure S1: SEM Images of representative fabricated metasurfaces. Top-view of some of the studied metasurfaces. From left to right, the first three show increasing angle α at fixed intra-cell distance parameter d. The last two compare the case of non-equidistant vs equidistant meta-atoms (d = 1.0) at fixed α. Red scale bar at the bottom-right corner, 600 nm.

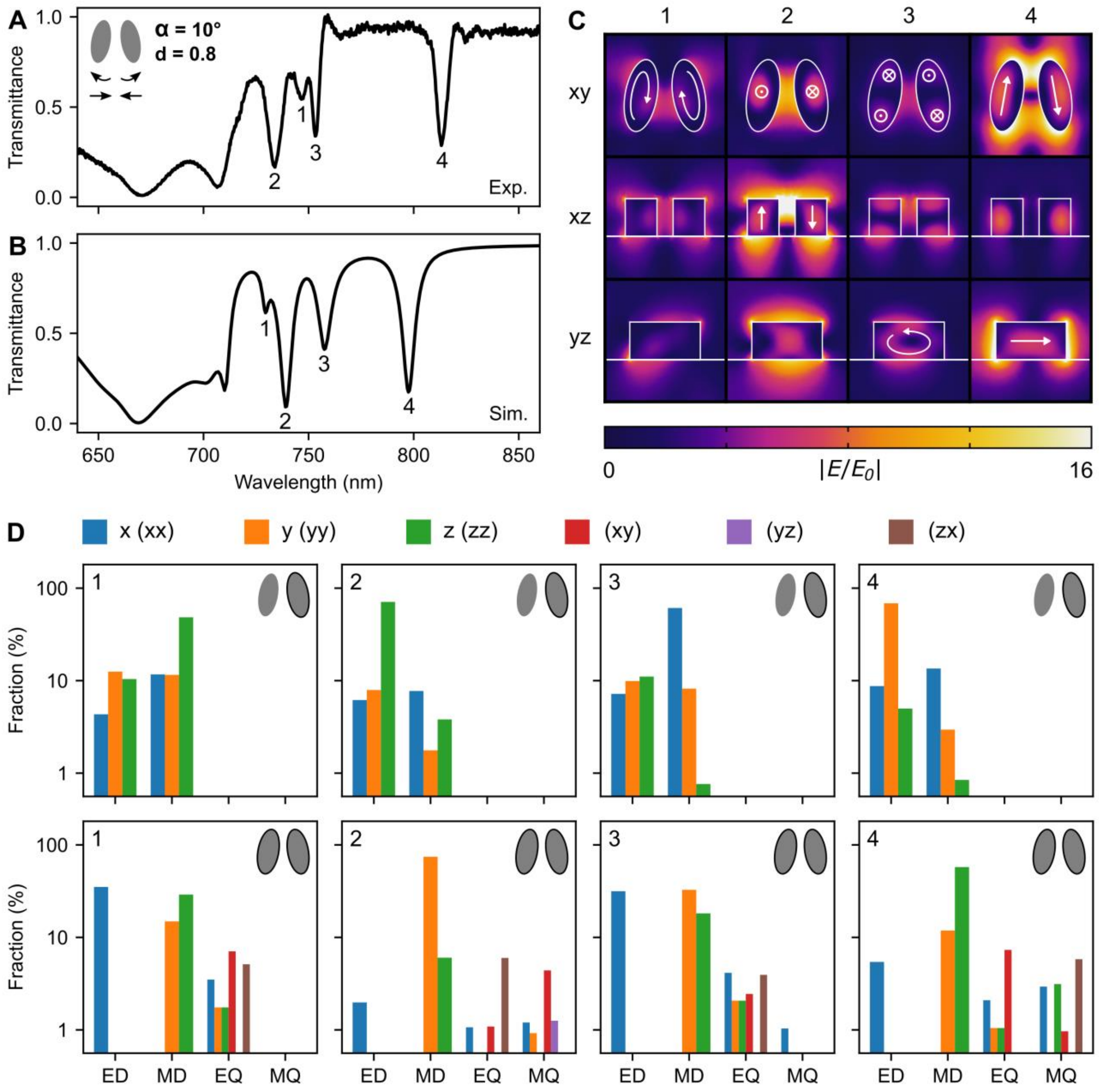

Figure S2: Field distribution and multipolar decomposition of all resonances. Experimental **(A)** and simulated **(B)** transmittance spectra for the d = 0.8, α = 10° metasurface design (Figure 3 of the manuscript), with the four states described in the main text labeled. **(C)** Electric field distributions, with each column corresponding to the resonance denoted at the top and each row to different cross-sections of the unit cell. **(D)** Electric dipole (ED), magnetic dipole (MD), electric quadrupole (EQ) and magnetic quadrupole (MQ) moments of the system with their cartesian fractions (x, y, z for dipoles and xx, yy, zz, xy, yz, zx for quadrupoles). Each column corresponds to the mode marked in the top-left corner of the bar-charts and each row to different integration volumes, marked as black outlines in the top-right corners. The resonance labeled as '4' is characterized by an out-of-plane (z) magnetic dipole and an in-plane (xy) electric quadrupole (second row, last column). When considering only one element of the dimer (first row, last column) a y-pointing electric dipole dominates. Resonance '2' (second row, second column) is described by an electric field circulation in the xz plane (magnetic dipole pointing in y). When contemplating an individual element of the unit cell (first row, second column), an electric field component normal (z) to the substrate governs. It is straightforward to see that resonances '1' and '3' are the magnetic counterparts of '2' and '4', respectively, when considering single meta-atoms. The first one (first row, first column) replaces the electric dipole in the z-direction with a magnetic dipole also in z, while the second one (first row, third column) presents a magnetic dipole in the x-direction instead of the y-pointing electric dipole.

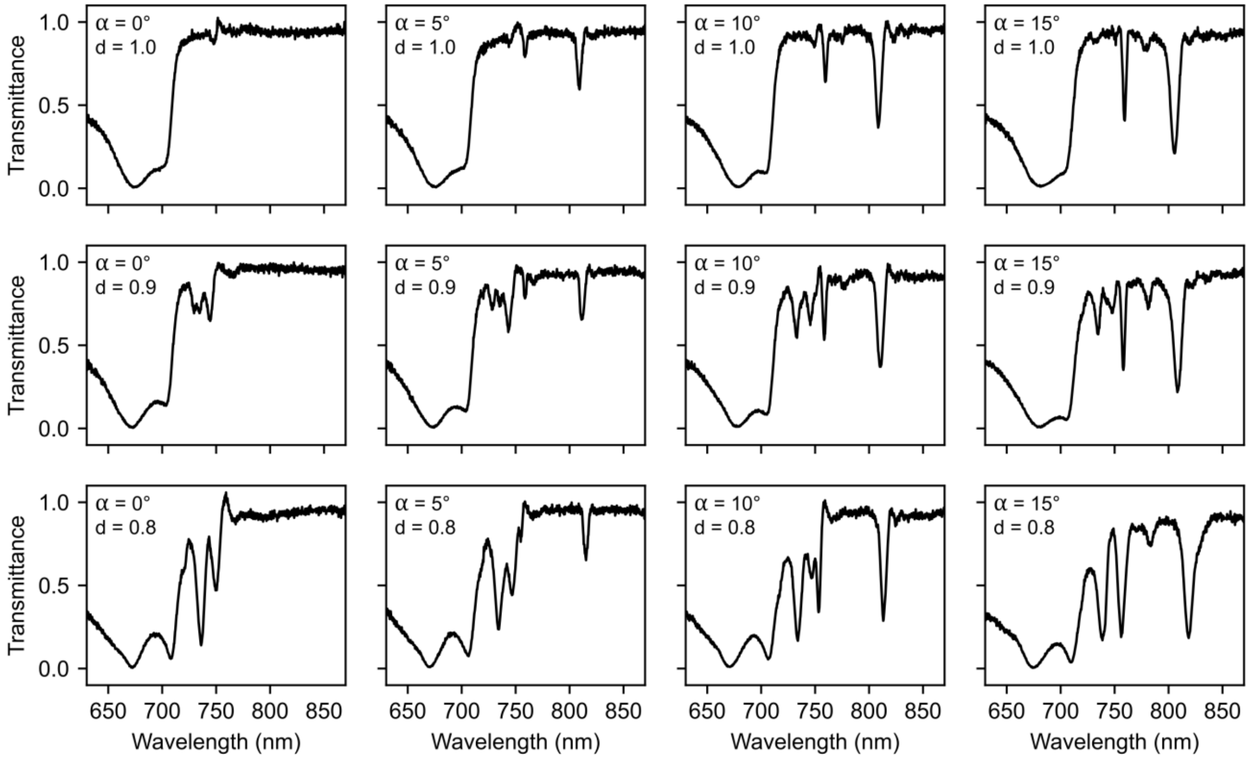

Figure S3: Additional transmittance spectra for varying α and d. Transmittance spectra of metasurfaces with α ranging from 0° to 15°, and d from 0.8 to 1. The top-left plot shows the fully symmetric metasurface, each successive column increases the angle, and each row below decreases the intra-cell meta-atom distance.

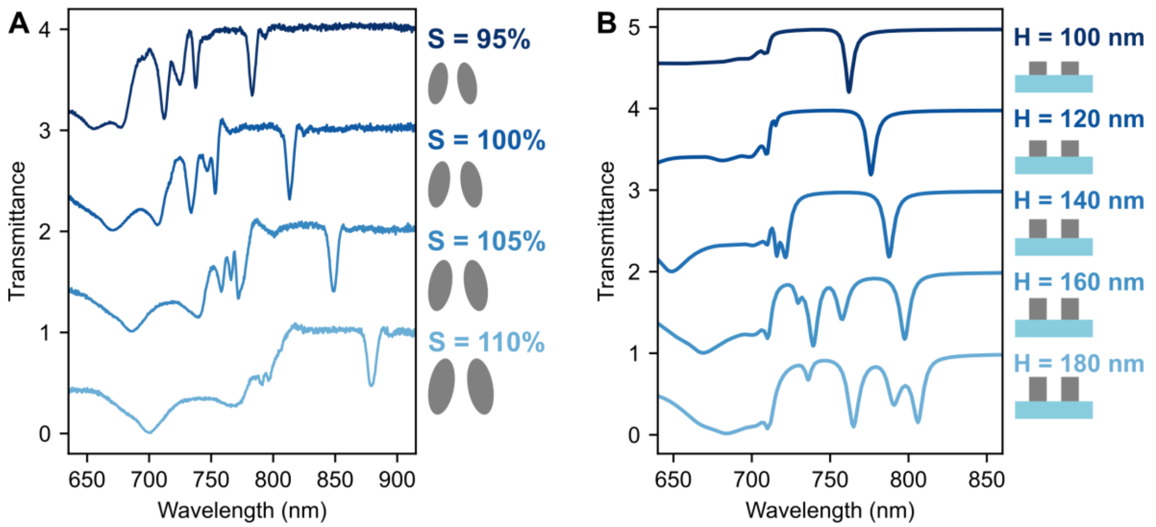

Figure S4: Results for different lateral dimensions and simulations changing the height. (A) Transmittance spectra for different sizes of the unit cell. The height is kept fixed at 160 nm, and the ellipses diameter and periodicity are scaled (S) up or down; S = 100% corresponds to the nominal design. **(B)** Simulated transmittance for different metasurface heights (displayed on the right of the graph) while keeping the rest of the geometrical dimensions constant. It can be seen how a minimum height is required to support more than one qBIC resonance. The transmittance spectra are vertically displaced for clarity.

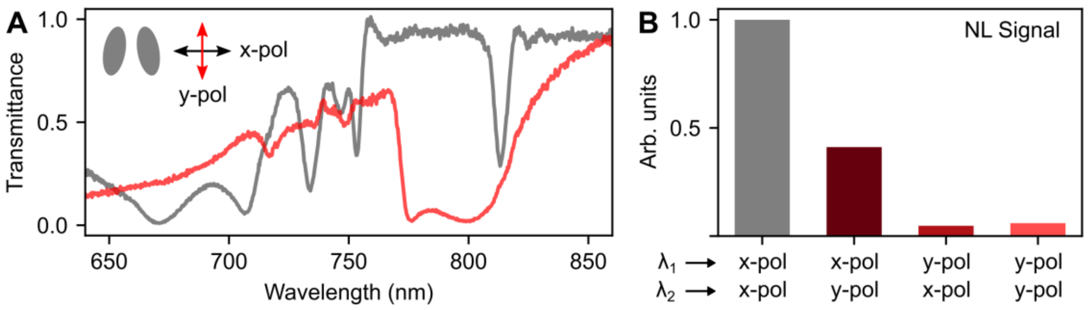

Figure S5: Linear and nonlinear experiments with y-polarized light. (A) Comparison of the transmission response with x-polarized and y-polarized light for the d = 0.8, α = 10° metasurface design. A scheme of the unit cell in the top-left corner shows the direction of the polarization with respect to the meta-atoms orientation. (B) Nonlinear signal at λ_1 = 741 nm and λ_2 = 816 nm (same as in Figure 4 of the manuscript) for different polarization conditions. The maximum signal is obtained when both pump lasers are x-polarized, and the minimum when the 'Pump 1' laser is y-polarized.

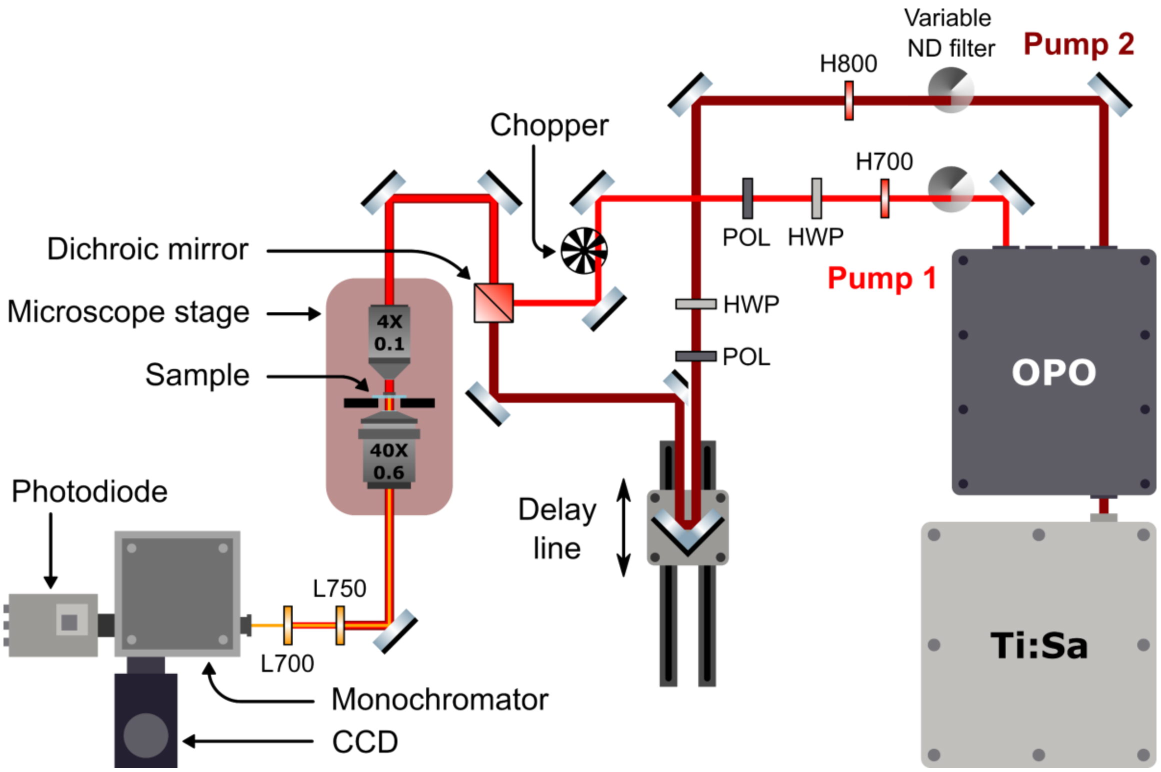

Figure S6: Experimental setup for nonlinear measurements. 'Pump 1' corresponds to the second harmonic of the OPO signal (500-800 nm) and 'Pump 2' to the Ti:Sapphire laser (700-1000 nm). Each arm before the sample has high-pass filters (H700 and H800), polarization optics (half-wave plates, HWP and linear polarizers, POL) and neutral density filters (ND). In this way the interference of the nonlinear signal with the tails of the lasers can be avoided and the polarization alignment and intensity on the sample is controlled. On the detection side two low-pass filters (L700 and L750) are used to measure the generated light at λ_s ~ 640 - 690 nm. The delay line is used to temporally overlap both laser pulses at the sample position.

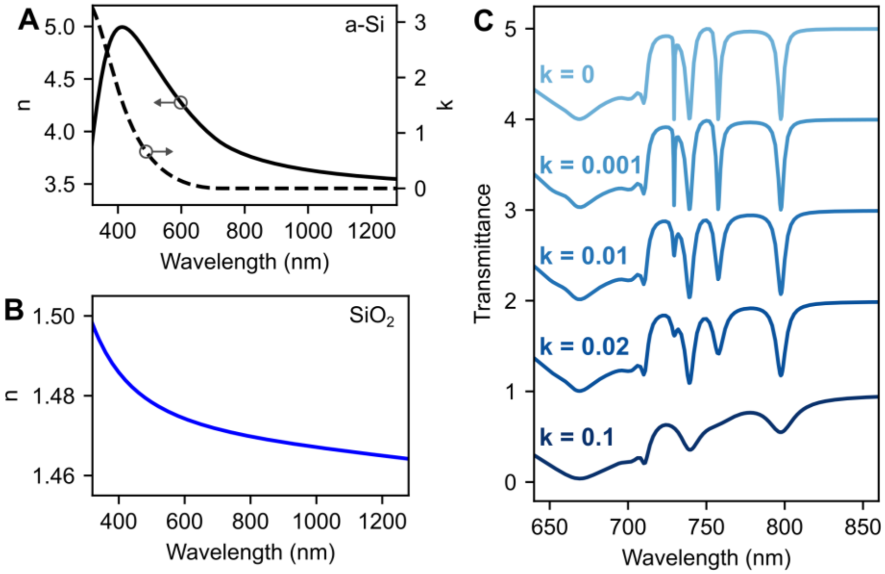

Figure S7: Ellipsometry data for Si and SiO_2 and simulations changing k. Refractive index (n) and extinction coefficient (k) for a-Si **(A)** and SiO_2 **(B)** obtained from ellipsometry measurements. **(C)** Simulations showing the transmittance spectra when increasing k, where a broadening of the resonances is observed. Although a-Si exhibits k < 0.001 above 700 nm, we considered a k value of 0.02 when comparing to experiments to better approximate other non-radiative losses, like surface roughness. The transmittance spectra are vertically displaced for clarity.
